# Crystal Structure and Characterization of Human Heavy-Chain Only Antibodies Reveals a Novel, Stable Dimeric Structure Similar to Monoclonal Antibodies

**DOI:** 10.3390/antib9040066

**Published:** 2020-11-22

**Authors:** Carl Mieczkowski, Soheila Bahmanjah, Yao Yu, Jeanne Baker, Gopalan Raghunathan, Daniela Tomazela, Mark Hsieh, Mark McCoy, Corey Strickland, Laurence Fayadat-Dilman

**Affiliations:** 1 Discovery Biologics, Protein Sciences, Merck & Co., Inc., South San Francisco, CA 94080, USA; yao.yu@merck.com (Y.Y.); jeanne.baker@merck.com (J.B.); gopalan.raghunathan@merck.com (G.R.); daniela.tomazela@merck.com (D.T.); mark_hsieh@merck.com (M.H.); laurence.fayadat-dilman@merck.com (L.F.-D.); 2 Department of Chemistry, Modeling and Informatics, Merck & Co., Inc., Kenilworth, NJ 07033, USA; soheila.bahmanjah@merck.com (S.B.); corey.strickland@merck.com (C.S.); 3 Department of Pharmacology, Mass Spectrometry & Biophysics, Merck & Co., Inc., Kenilworth, NJ 07033, USA; mark.mccoy@merck.com

**Keywords:** antibody, heavy-chain dimer, heavy-chain antibody, crystal structure

## Abstract

We report the novel crystal structure and characterization of symmetrical, homodimeric humanized heavy-chain-only antibodies or dimers (HC2s). HC2s were found to be significantly coexpressed and secreted along with mAbs from transient CHO HC/LC cotransfection, resulting in an unacceptable mAb developability attribute. Expression of full-length HC2s in the absence of LC followed by purification resulted in HC2s with high purity and thermal stability similar to conventional mAbs. The V_H_ and C_H_1 portion of the heavy chain (or Fd) was also efficiently expressed and yielded a stable, covalent, and reducible dimer (Fd2). Mutagenesis of all heavy chain cysteines involved in disulfide bond formation revealed that Fd2 intermolecular disulfide formation was similar to Fabs and elucidated requirements for Fd2 folding and expression. For one HC2, we solved the crystal structure of the Fd2 domain to 2.9 Å, revealing a highly symmetrical homodimer that is structurally similar to Fabs and is mediated by conserved (C_H_1) and variable (V_H_) contacts with all CDRs positioned outward for target binding. Interfacial dimer contacts revealed by the crystal structure were mutated for two HC2s and were found to dramatically affect HC2 formation while maintaining mAb bioactivity, offering a potential means to modulate novel HC2 formation through engineering. These findings indicate that human heavy-chain dimers can be secreted efficiently in the absence of light chains, may show good physicochemical properties and stability, are structurally similar to Fabs, offer insights into their mechanism of formation, and may be amenable as a novel therapeutic modality.

## 1. Introduction

Typical monoclonal antibodies (mAbs) with specificity towards a target antigen are composed of heavy (HC) and light (LC) chains containing conserved and variable regions. Previously, heavy- chain only antibody (HCAb) formation was reported to occur in various species with significant human therapeutic potential [1]. Camelids are long known to express functional HC-only antibodies that are composed of a homodimeric V_HH_ domain [2,3]. Further, sharks produce functional heavy-chain only antibodies, that like camelid antibodies, are smaller in nature, and formed the basis of nanobody technology [4,5]. Like camelid V_HH_ domains and shark nanobodies, both lacking C_H_1 and LC domains, HCAbs have been reported to be secreted in LC-deficient mice lacking the C_H_1 domain [6]. Separately, hybrid llama/human antibody HCAbs, lacking the C_H_1 domain and having swapped the llama V_HH_ regions with human V_H_, have been reported [7]. In addition, HC-only transcripts, lacking the C_H_1 domain and in the absence of LC, can be expressed on the cell surface of mammalian pro-B cells [8]. What is noteworthy here with these examples of HC-only antibodies found in camelids, sharks, LC-deficient mice, and mammalian pro-B cells is that the presence of these molecules does not contradict the longstanding views on antibody mAb or Fab assembly, where LC assembly to the HC, or in particular to the C_H_1 domain, is required for C_H_1 domain folding and dissociation from the molecular chaperone BiP [9,10,11]. Interestingly, it has been reported that full- length HC-only antibody dimers are formed from a stable Drosophila cell line via a BiP mediated pathway [12]. This observation challenges the long-held hypothesis that the unfolded C_H_1 domain in complex to the molecular chaperone BiP requires association with LC to fold and release BiP chaperone, enabling export and secretion. Nonetheless, the formation of full-length HC-only antibodies is uncommon, and aside from the normal requirement of the LC to bind chaperoned C_H_1 and release BiP, additional mechanisms may be required to neutralize their potential toxicity in the absence of LC as previously reported in plasma cells [13].

Human HCAbs have only recently been reported by Stoyle and coworkers to occur from transient Chinese Hamster Ovary (CHO) expression [14]. Like antibody producing B cells, CHO cells have a similar quality control system and mechanism of antibody assembly, utilizing BiP, prolyl isomerases, and disulfide reductases [15]. Therein, HCAbs containing the constant C_H_1 and V_H_ regions humanized from rodent sources were found to form homodimers and be secreted even in the absence of light chain. These HC dimers were found to form from both HC/LC cotransfected cells and HC-only transfection, and both full-length HC dimers and HC dimers lacking the Fc domain (V_H_ + C_H_1 only) were able to form. The LC-independent secretion of HC dimers was inferred to be variable region dependent since only certain HCs were able to form and be secreted as folded molecules. One characteristic noted for some of the molecules being able to form HC2s was the increased number of positively charged amino acids in the HC-CDR3 [14].

Although Stoyle and coworkers clearly showed that both full-length heavy-chain dimers (HC2s) and HCs lacking the Fc domain (analogous to the Fab domain, herein described as “Fd2”) are able to be formed and secreted in the absence of LC, it is not clear what their mechanism of assembly is, nor is it clear what are the sequence and structural determinants that drive their formation. Also lacking are biophysical and structural characterizations of these novel molecules. The formation of human HC2s is unique and offers extraordinary insights into antibody assembly, as well as potentially enabling different, novel antibody formats with unique advantages and properties. One potential advantage is that common LC mispairing in multi-specific formats can be avoided. However, without further understanding their biophysical properties and how structural and sequence elements impact their formation, engineering and modulating HC dimers into a novel potential modality cannot be achieved. Aside from assumed differences in the HC variable sequence that impact HC2 formation, the role of the LC is also not well understood and how this competing pathway to normal HC-LC assembly could impact HC-HC (HC2) assembly.

Herein, we report robust expression and formation of human HC2s for two different humanized antibodies with unrelated variable sequences against different biological targets. Robust formation of HC2 impurities originally constituted a severe, negative mAb developability attribute. In HC/LC cotransfected transient CHO cells, HC2 formation was robust and prevented further developability of these clones as acceptable purity/heterogeneity of the desired mAb species was unattainable under representative process conditions. To characterize further these novel and unique “impurities”, we performed a series of HC-only transfections and characterized the secreted and purified HC dimers. For both full-length and Fd2 versions, we found these molecules can achieve high purity, and to have similar or better expression titer, thermal stability, and accelerated storage stability than their mAb and Fab counterparts, respectively. Additionally, we solved the first ever crystal structure at 2.9 Angstroms (Å) of a human HC homodimer (herein named Fd2-A) that reveals exposed CDR regions and a symmetrical dimerization complex analogous to HC-LC association in Fabs, where one opposing HC (C_H_1 + V_H_ or Fd) replaces the LC. Disulfide formation is overall conserved between this Fd2 complex in comparison to the Fd domain in Fabs. Mutagenesis of key, variable CDR residues in the dimerization interface indicated by the novel Fd2-A crystal structure reduced full-length HC dimer formation. Similar mutations were made for a second recombinant antibody based on a sequence alignment to Fd2-A and similar HC2 dimer reduction was achieved, suggesting that this heavy-chain only dimer structure is conserved for two antibody sequences with different germlines against two different biological targets. These results indicate that we have solved a novel crystal structure representing the human HC dimer (Fd2) structure, provided new insights into their formation, structural and sequence determinants, and demonstrated that their application as a novel and functional antibody format is realistic.

## 2. Materials and Methods

### 2.1. Transient Protein Expression

For the small-scale protein production, transient transfections were done in TubeSpin^®^ bioreactors (TPP Techno Plastic Products AG, Trasadingen, Switzerland) using the ExpiCHO Expression System (Thermo Fisher Scientific, Waltham, MA, USA) according to the manufacturer’s protocol. Briefly, the cells were grown and maintained in ExpiCHO Expression Medium (Thermo Fisher Scientific, Waltham, MA, USA) and seeded in 10 mL of media at 6 × 10^6^ cells/mL on the day of transfection. Complexes were formed with 8 ug of DNA and 32 μL of Expifectamine in OptiPRO™ SFM and incubated for 1 min followed by addition to the cells. The transfected cultures were grown at 37 °C, 5% CO_2_, 80% humidity, and 300 rpm rotation in a Multitron incubator (Infors HT, Basel, Switzerland) and then shifted to 32 °C 24 h post-transfection and were fed with feed and enhancer on days 1 and 5. Expression variables include no enhancer treatment (Thermo), pulling culture on Day 4, using 20% of coding DNA by weight, transfecting with 5X LC by molar mass, and using a 10 mL shake flask instead of tubespin cultures. The cultures were harvested on day 7, the cells were pelleted by centrifugation, and the supernatant was passed through a 0.2 micron filter. The protein titers were determined using a ForteBio Octet (Molecular Devices, LLC. San Jose, CA, USA) with Protein A sensors and a purified mAb to generate the standard curve.

For large-scale protein production, transient transfections were performed in 1 L shake flasks using the ExpiCHO Expression System (Thermo Fisher Scientific, Waltham, MA, USA) according to the manufacturer’s protocol. Transfections and expression conditions were the same as in small-scale format. The cultures were harvested on day 7, the cells were pelleted by centrifugation, and the supernatant was passed through a 0.2 micron filter. The protein titers were determined using a ForteBio Octet (Molecular Devices, LLC. San Jose, CA, USA) with Protein A sensors and a purified mAb to generate the standard curve.

### 2.2. Small-Scale Protein Purification

The clarified cell culture supernatants were loaded onto a Tecan Freedom EVO 200 (Tecan Life Sciences, Männedorf, Switzerland) for antibody purification utilizing miniature columns manufactured by Repligen (Waltham, MA, USA) and packed with MabSelect™ SuRe™ LX (GE Healthcare Life Sciences, Pittsburgh, PA, USA). The antibodies were eluted with 20 mM sodium acetate at pH 3.5 and immediately neutralized with 0.33 M Tris, 1 M sodium acetate pH 8.0 and buffer exchanged into 20 mM sodium acetate pH 5.5 using 10K MWCO Slide-A-Lyzer dialysis cassettes (Thermo Fisher Scientific, Waltham, MA, USA).

### 2.3. Large-Scale mAb and Full-Length HC Dimer Purification

Cell culture supernatant was incubated with Protein A (ProA) affinity MabSelect SuRe LX resin (GE Healthcare, Pittsburgh, PA, USA) overnight (1 mL resin for 40 mg target antibody or HC dimer estimated by ForteBio Octet) for batch binding. Affinity resin with bound protein was collected by filtration and transferred to a disposable column. Bound resin was washed with 20X column volumes of 1X Gibco Phosphate buffered saline (PBS) (Thermo Fisher Scientific, Waltham, MA, USA) in batch mode. Desired protein was eluted by approximately 5 column volumes of 20 mM sodium acetate pH 3.5 buffer. Eluate was immediately buffer exchanged into 20 mM sodium acetate pH 5.5 buffer using 10K MWCO Slide-A-Lyzer dialysis cassettes. To further polish material for analytical and biophysical characterization efforts (DSC, SEC-MALS, LC-MS), desired product was purified to >98% purity (by SE-UPLC and cSDS) on a Superdex 200 Increase 10/300GL column (GE Healthcare Bio-Science AB, Uppsala, Sweden). Mobile phase was 20 mM sodium acetate, 200 mM sodium chloride pH 5.5 buffer. All purified protein was buffer exchanged overnight using 10K MWCO Slide-A-Lyzer dialysis cassettes into 1X Gibco PBS pH 7.4 and normalized to 1 mg/mL for characterization. Concentration was determined by UV absorbance at 280 nm on a Nanodrop 2000 1- position Spectrophotometer (Thermo Scientific).

### 2.4. Fab and Fd Dimer Purification

Cell culture supernatant was incubated with CaptureSelect IgG-C_H_1 affinity matrix (Thermo Scientific) overnight for batch binding (1 mL resin for 10 mg protein estimated by ForteBio Octet). Resin was collected by filtration, transferred to a disposable column, and then washed with 20 column volumes of 1X Gibco PBS in batch mode. Desired protein was eluted with 5 column volumes of 0.1 M glycine pH 3.0 buffer. Eluate was immediately buffer exchanged into 20 mM sodium acetate pH 5.5 buffer. To further polish material for biophysical characterization and crystallization efforts, desired product was purified to >98% purity (by SE-UPLC and cSDS) on a Superdex 200 increase 10/300GL (GE Healthcare Bio-Science AB, Uppsala, Sweden) column. Mobile phase was 20 mM sodium acetate, 200 mM sodium chloride pH 5.5 buffer. All purified protein was buffer exchanged overnight into 1X Gibco PBS pH 7.4 and normalized to 1 mg/mL for characterization. For crystallization efforts, Fd2 for Molecule A was reformulated into 20 mM sodium acetate pH 5.5 (low salt) and concentrated to 20 mg/mL using a 4 mL Vivaspin™ ultrafiltration spin column with 10K MWCO membrane. Purity was verified to be unchanged following concentration by SE-UPLC.

### 2.5. Size-Exclusion Ultra-Pressure Liquid Chromatography (SE-UPLC)

A Waters Acquity UPLC H-Class PLUS system (Waters, Milford, MA, USA) was used to separate molecules based on differences in their hydrodynamic size. Samples (10 μg) were injected into an Acquity BEH200 SEC column (Waters, Milford, MA, USA) and eluted at a 0.5 mL/min flow rate. Mobile phase contained 100 mM sodium phosphate and 200 mM sodium chloride pH 7.0. Waters BEH200 SEC protein standard mix (Waters, Milford, MA, USA) was used as molecular weight marker and for column quality control purposes. Samples were detected by UV absorbance at 280 nm. Chromatograms were integrated manually and reported as a % of integrated area for each species.

### 2.6. Capillary Sodium Dodecyl-Sulfate Electrophoresis (cSDS)

A LabChip GXII Clipper (Perkin Elmer, Waltham, MA, USA) was used to determine purity and approximate molecular weight under non-reduced and reduced conditions. All sample and chip preparation was done according to manufacturer’s protocol using the Protein Express Assay reagent kit (Perkin Elmer). Five μL of sample was mixed with 35 μL of reduced Protein Express Sample Buffer (35 mM Dithiothreitol or DTT added to kit sample buffer) or 35 μL non-reduced sample buffer (35 mM iodoacetamide added to kit sample buffer). An HT Protein Express Assay LabChip (Perkin Elmer) was primed using the Protein Express Assay Reagent kit (Perkin Elmer). LabChip GX Reviewer software was used for data analysis. Chromatograms were integrated manually and reported as % for each species. This method produces system or method-related peaks that appear at before 10 KDa in size and include reagents and the internal 10 KDa molecular weight standard.

### 2.7. Size Exclusion Chromatography Coupled to Multi-Angle Light Scattering (SEC-MALS)

A µDAWN (Wyatt Technology, Santa Barbara, CA, USA) was coupled online to a Waters UPLC H-Class system (Waters, Milford, MA, USA) to measure molecular weight (Mw) using static light scattering. Light scattering wavelength used was 650 nm. The concentration detector was an online uTrex equipped with a refractive index detector. Dn/Dc value used in Mw calculations was 0.185 mL/g. PBS was used as the mobile phase and 20 μg samples were injected. Other SE-UPLC running conditions used are described here in the SE-UPLC methods section. Bovine serum albumin (Thermo Scientific) was used as an isotropic standard for molecular weight normalization. Astra 7 software (Wyatt Technology, Santa Barbara, CA, USA) was used for data acquisition and analysis. Detector alignment, band broadening, and normalization coefficient parameters were set to achieve a BSA Mw within 5% of 66,500 Da.

### 2.8. Differential Scanning Calorimetry (DSC)

A MicroCal PEAQ-DSC (Malvern Panalytical, Malvern, UK) was used to measure protein melting temperatures. An amount of 500 μL of protein solution for each sample at 1 mg/mL in 20 mM sodium acetate pH 5.5 was added to a 96-well 500 μL volume plate (Wheaton). Temperature was ramped from 25 °C to 95 °C at 1 °C per min. Origin 7 software was used for data acquisition and analysis where melting transition temperatures (T_m_) were fitted using a non-two state algorithm.

### 2.9. Nanoscale-Differential Scanning Fluorimetry (Nano-DSF)

All nano-DSF studies were performed using the Nanotemper Prometheus NT.48 instrument and data analysis software. Samples were evaluated at 1 mg/mL in 20 mM sodium acetate pH 5.5. Samples were introduced by capillary action into glass capillaries (Prometheus) prior to placing into the instrument capillary holder. Temperature was ramped from 20 °C to 95 °C at 1 °C/minute. Thermal melting transitions (T_m_) were measured by identifying changes in inflection of intrinsic fluorescence intensity ratios (F350 nm/F330 nm) at specific temperatures.

### 2.10. Surface Plasmon Resonance (SPR) Affinity Measurements by BIAcore

Binding kinetics of the mAbs to the target was determined by SPR on a BIAcore T200 (GE Healthcare, Chicago, IL, USA). The running buffer, 10 mM HEPES, 150 mM NaCl, 0.05% (*v*/*v*) Surfactant P20, 3 mM EDTA, pH 7.4 (HBS-EP+, GE Healthcare), was used for immobilization and reagent dilutions. All binding kinetics were measured at 25 °C. For each injection cycle, mAbs were first captured in different flow cells with an anti-human Fc antibody (Human Antibody Capture Kit, GE Healthcare) immobilized to the sensor chip (Series S CM5, GE Healthcare). Reference flow cell with no captured mAb was also used. Serial dilutions (1:2) of the target protein, ranging in concentration from 1 μM to 32 μM, and buffer blanks were injected in multiple cycles over the captured mAbs and reference surfaces for a 1-min association followed by a 3-min dissociation. The surfaces were regenerated with a 30 s injection of 3 M MgCl2 after each cycle. Double-referenced titration data was globally fit to a 1:1 Langmuir binding model to determine the association rate constant, ka (M−1 s−1), and the dissociation rate constant, kd (s−1), using the BIAcore T200 Evaluation Software version 2.0 (GE Healthcare). The equilibrium dissociation constant was calculated as KD (M) = kd/ka.

### 2.11. Liquid Chromatography Mass Spectrometry (LC-MS or Intact Mass)

Sample was diluted to 0.2 mg/mL with 50 mM ammonium bicarbonate and 4 μL was injected to a POROS R2/10 2.1 × 30 mm Column (Life Technologies, Carlsbad, CA, USA, 1-1112-12). A gradient from 30 to 58% Buffer B (acetonitrile, 0.1% formic acid) in Buffer A (water, 0.1% formic acid) was applied to the chromatographic column with flow rate of 100 μL/min. Data was acquired on a Waters Synapt G2-S Mass Spectrometer and deconvoluted to monoisotopic and singly-charged using the Waters MassEnt 1 software.

### 2.12. Storage Stability Study

All samples were formulated in 20 mM sodium acetate pH 5.5 at 5 mg/mL. Solutions were filtered through a Millex-GV 0.2 μM PVDF 33 mm syringe filter (Millipore, Burlington, MA, USA) and aliquoted into 2 mL screw-top microcentrifuge tubes (Fisher). Samples subjected to accelerated storage were placed into a temperature-controlled stability chamber (Thermo Scientific) at 40 °C. Samples were pulled at specific timepoints and then analyzed for purity by SE-UPLC and cSDS.

### 2.13. Crystallization and Data Collection

Crystallization plates were set up in 3 sub-well plates (Intelli, Art Robbins) by vapor diffusion using Mosquito (TTP Labtech, Boston, MA, USA) at 4, 18, and 30 °C, and images were acquired using RockImager 1000 (Formulatrix Bedford, MA, USA). Crystals appeared in well of H7 of JCSG Plus Screen (Molecular Dimensions, Maumee, OH, USA) within a few hours and were fully grown after 3 days. Crystals (length, 80–200 μm) were present in condition H7 (0.2 M ammonium sulfate, 0.1 M Bis-Tris pH 5.5, 25% *w/v* PEG 3350) in 1:1, 2:1 and 1:2 protein to precipitant ratio in 200 nL drops. Further optimization of condition resulted in optimal crystal in 2:1 protein to precipitant ratio at 30 °C of 200 nL drops. Crystals were cryo-protected in reservoir solution supplemented with 5% glycerol and flash-cooled in liquid nitrogen. We noticed that crystals harvested after 2–3 days resulted in optimal diffraction. Data collection was performed at the Industrial Macromolecular Crystallography Association (IMCA) beam line, sector 17 of the Advanced Photon Source (APS) at the Argonne National Laboratory (ANL, Lemont, IL, USA). Data were collected at a wavelength of 1.0 Å using a Pilatus 6M detector (Dectris A G, Baden Dättwil, Switzerland). The data were processed using the autoPROC [16,17] automated processing software. AutoPROC utilizes XDS for indexing and integration and, AIMLESS for scaling, POINT LESS for data analysis, and STARANISO for applying anisotropic diffraction limits.

### 2.14. Structure Determination and Model Building

The structure was solved by Molecular Replacement using MOLREP [18] and Phaser [19]. The partial model was further extended by AutoBuild [20]. The structure was then refined using autoBUSTER [21] and phenix.refine [22]. The initial maps had poor density for several regions, including some of the CDR-like loops, which were removed from the model and gradually rebuilt during refinement. The electron density map was consistent with most sequence substitutions and insertions or deletions between the starting molecular replacement model and the final structure. The sequence was manually corrected using COOT [23]. The resulting structure was refined using Phenix and rebuilt several times leading to final values of R_free_ and R_work_. The final model contained 3 dimers in the asymmetric unit.

### 2.15. Constructs Used, Sequence Alignment, and Numbering

All mAbs, Fabs, HC2s, and Fd2 molecules used in this study were prepared by gene synthesis. Full length mAb versions contained all residues within the V_H_ and CH domains. mAbs A and B were humanized from mouse and rat immunization campaigns, respectively. For full-length HC2 molecules, the entire HC was used without alterations or truncations. Mutated HC2 molecules were prepared by site-directed mutagenesis. For design of Fabs and Fd2s, engineered IgG1 HC constructs were terminated just prior to the hinge region (or residues 1-224 for Fab-A and Fd2-A and residues 1-235 for Fab-B and Fd2-B) ending in the conserved sequence THT. For the LC, the full-length LC sequence was used (residues 1-219 for Fab-A and residues 1-213 for Fab-B). Both LC sequences terminated in the conserved cysteine involved in the HC:LC intermolecular disulfide. Sequences were aligned and CDRs annotated using proprietary Abacus^TM^ Antibody & Engineering Analysis software using standard pre-sets and reference antibody sets. Default numbering referenced throughout the text is based on sequential numbering, with the exception of the sequence alignments generated by Abacus^TM^, where default sequential numbering was used throughout the alignment by the software.

## 3. Results

### 3.1. Observation and Identification of Expressed and Purified Humanized HC Dimer

**Figure 1:**
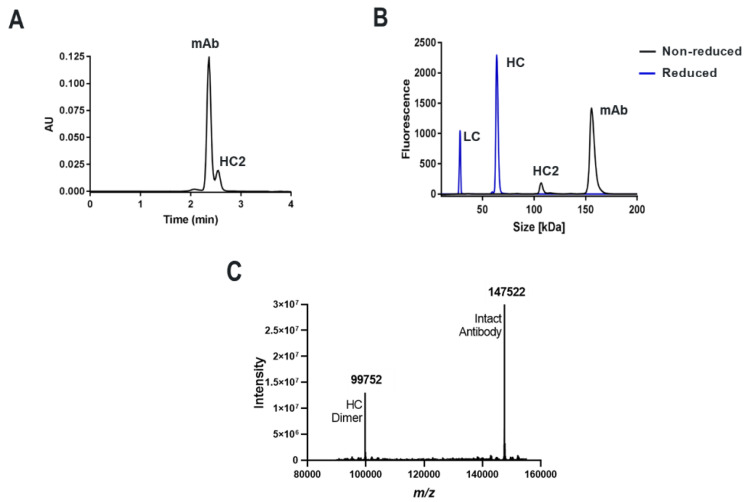
Detection of HC2 species in humanized mAb-A molecule by (**A**) SE-UPLC and (**B**) NR- and Red-cSDS (profiles overlaid). (**C**) Confirmation of HC2-A species by intact mass spectrometry from a mixture of mAb and HC2 species.

Humanized heavy-chain dimer (HC2) formation was observed for two different monoclonal antibodies from transient HC/LC cotransfection followed by Protein A affinity capture. For a recombinant IgG1 mAb, designated here as “mAb-A”, a species corresponding to an HC:HC homodimer complex was apparent by NR-cSDS and a lower molecular weight species was partially resolved by SE-UPLC (Figure 1). No other species aside from expected HC and LC were detected by Red-cSDS, although a higher ratio of HC:LC was observed. In Figure 1, representative SE-UPLC and cSDS profiles are shown for mAb-A (Figure 1A,B respectively), along with the verification of covalent full-length HC dimer by intact mass spectrometry (Figure 1C). By NR-cSDS, the full-length IgG1 HC2 species was measured at 4.9% for a standard tube spin 10 mL culture expression (See Table 1 for data). This species is designated “HC2-A”. Other constructs corresponding to this same molecule, such as the Fab, Fd portion (C_H_1 + V_H_), or full-length mAb, are designated “Fab-A”, “Fd-A”, “mAb- A”, and so on, deriving from the parent molecule A full-length mAb construct. For other molecules evaluated herein (such as B, C, and D), the same nomenclature applies.

### 3.2. Effect of Expression Variables on HC Dimer Expression

Initially, it was not clear to us what were the root causes of HC dimer formation; specifically, if it was artifactual expression or an inherent property of the antibody sequence driving its assembly. Therefore, we investigated the effects of isotype (human IgG1 versus IgG4), expression variables and DNA codon use on HC2 formation. mAb-A was evaluated as IgG1 and IgG4 (S228P) [24] isotypes alongside a negative control mAb known not to form HC2s. This control is a typical recombinant mAb used as an expression control known for its robust titers and yield. Expression variables such as shaking method, enhancer addition, culture time, and codon optimization were explored for both IgG1 and IgG4 isotypes containing the same variable sequence, and levels of HC2 were quantified by cSDS. In Table 1, these expression conditions are summarized for all molecules across all expression conditions for HC/LC cotransfection and expression. For the controlled “tube spin” condition, both HC-only transfection and HC/LC cotransfection were evaluated. In a 10 mL tube spin transient culture from HC- only transfection, greater than 1 mg of HC2 was captured (or >100 mg/L yield from culture supernatant) for both IgG1 and IgG4 isotypes at a purity of ≥98% by NR-cSDS. Therefore, HC-only expression of mAb-A resulted in efficient expression of HC2 and was affinity captured at high purity. For HC/LC cotransfections, the percent of HC2 quantified by NR-cSDS was approximately 5–7% for both isotypes and ranged from 1.6% to 15.7% across all expression conditions and codon variations, indicating that HC dimer levels are somewhat variable but also robust across experimental conditions. Therefore, it was clear that HC dimer formation was not a consequence of a particular expression or process-related artifact. Our further characterization and investigation of these novel HC2 domains thus utilized the IgG1 isotype arising from standard expression conditions and standard purification.

### 3.3. Small-Scale Expression of Other Recombinant mAbs and HC Dimer Occurrence

Using 10 mL small-scale transient tube spin cultures, 12 additional random recombinant mAbs against unique targets were expressed as HC-only transfections and <0.1 mg HC2 or total protein was Pro-A captured for 11 out of 13 (mAbs-C to -M, Table 2). For all the molecules designated C-M that were expressed as HC/LC (mAb) cotransfections, <0.1% potential HC dimer was quantified by NR- CSDS. However, mAb-B was found to efficiently express HC2 in both HC/LC cotransfections and HC-only transfections. Therefore, as expected, HC dimer formation was unique to certain antibody molecules and sequences and not a common or frequent phenomenon across various antibody sequences. In Table 2, the HC/LC germline families are also tabulated for each mAb. Four of the total 13 sequences are considered rare or uncommon (<10 occurrences out of 358 sequences containing kappa light chains evaluated in Jayaram, et al.), and are highlighted red [25]. Of these four, two express this unique HC dimer at significant levels for both mAb and HC-only expression; specifically, mAbs A and B. The other nine molecules that do not significantly form HC2 all have common or frequently arising germlines (>20 occurrences out of 358 antibody sequences containing kappa light chain evaluated).

### 3.4. Large-Scale HC-Only Expression of 4 mAbs

At one liter or greater scale in CHO transient transfection, we expressed multiple full-length IgG1 HC antibody sequences designated as HC2-A to -D (Table 3) and ProA affinity captured the HC2 molecule of interest. Like HC2-A, HC2-B efficiently expresses as an HC dimer and even higher levels of HC2 are observed from HC+LC cotransfection (46.2%, See Table 2). Following HC-only transfection and expression, both HC2-A and HC2-B also were ProA affinity captured to relatively high purity and yield. In Table 3, HC2-A and HC2-B were expressed and ProA captured with a yield of 61 mg/L and 103 mg/mL respectively, from simply an unoptimized transient CHO expression, levels that are quite typical for transiently expressed mAbs. By NR-cSDS, purities were measured at 93.8% and 91.2% respectively for HC2-A and HC2-B, and greater than 96% by Red-cSDS for both. SE- UPLC and cSDS purities were increased to >98% following size-exclusion purification (data not shown) for further characterization, storage stability, and crystallization. Two other molecules (HC2- C and HC2-D) with little propensity to form HC dimer were also evaluated. Recall the mAb forms for these molecules exhibited <0.1% potential HC dimer in small-scale 10 mL HC/LC cotransfection (Table 2). For these two molecules that were HC-only transfected and expressed at large-scale, low amounts of HC dimer were observed at worse purity/heterogeneity, specifically less than 70% by NR-cSDS for HC2-D, indicating that other mAbs may be able to form low levels of lower quality HC2 from HC-only transfection at large-scale, but at insignificant levels during HC/LC cotransfection.

### 3.5. Accelerated Storage Stability of an HC2 Compared to mAb

To evaluate whether a full-length HC dimer has good storage stability and potential developability, we evaluated ProA/SEC purified full-length IgG1 mAb-A and HC2-A side-by-side on accelerated one-month storage at 40 °C and surveyed common degradants such as aggregation and fragmentation. Storage stability is a critical aspect of drug product development, and accelerated storage stability at elevated temperatures typically forecasts long-term storage at normal temperatures [26]. In Table 4, the % NR-cSDS purities and % SE-UPLC purities are tabulated at initial, 2 week, and 4 week timepoints for both mAb-A and HC2-A. The % of high molecular weight species quantified by SE-UPLC is also tabulated. NR-cSDS analysis was performed in order to mainly assess fragmentation and SE-UPLC was performed to assess both aggregation (HMW species formation) as well as potential LMW species or fragments. Remarkably, after 4 weeks at 40 °C, HC2-A has better NR-cSDS and SE-UPLC purity attributes versus the full-length mAb-A; by NR-cSDS and SE-UPLC, mAb-A had 96.1% and 98.7% purity, while HC2-A had 98.1% and 99.2%, respectively. Both mAb-A and HC2-A had similar and low levels of SE-UPLC HMW species or aggregation. These findings clearly indicate that HC2 antibodies may have excellent stability comparable to or better than mAbs.

### 3.6. Analysis of Two Fd (V_H_ + C_H_1) Domains (Fd2-A and Fd2-B)

**Figure 2:**
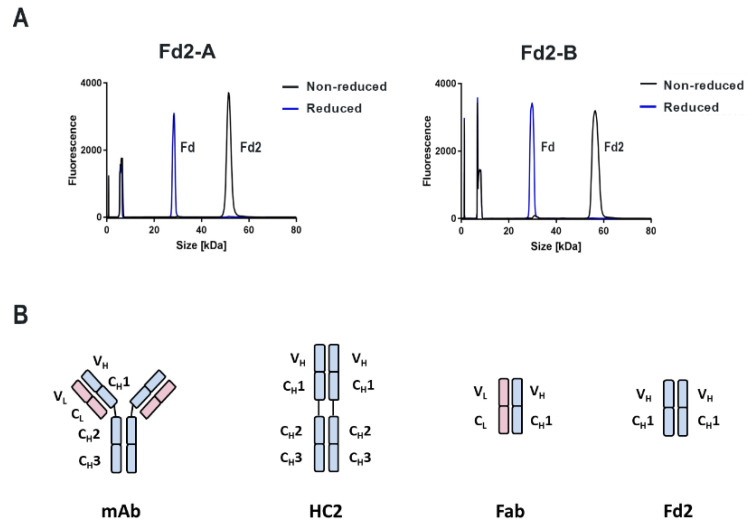
(**A**) Overlaid NR-cSDS and Red-cSDS profiles for both Fd2 forms Fd2-A and Fd2-B. The intact Fd2 dimer is reducible into monomeric Fd domains by DTT and appears as a Fd monomer by Red-cSDS. Peaks below approximately 10 KDa are system-related peaks and include the internal MW standard. (**B**) Diagrams representing a mAb, HC2, Fab, and Fd2. The HC is colored light blue and the light chain is light red. Each individual variable and constant domain composing the HC and LC are labeled. Intermolecular disulfides are not shown but exist in the hinge region and between C_L_ and C_H_1 as well as between the C_H_1 domains in the HC-only structures. HC-only Fd2 domains (V_H_ + C_H_1) are shown here to be associated.

To determine if HC2-A and HC2-B, which were shown to efficiently express full-length HC2 from both transient HC/LC cotransfection and HC-only transfection, can also form HC dimers using only the variable V_H_ and constant C_H_1 segment with no hinge and Fc domain present, the “Fd” (V_H_ + C_H_1) domain was expressed (designated as dimeric Fd2-A and Fd2-B as follows). These constructs were designed to end just prior to the IgG1 hinge, which contains two intermolecular disulfides. Therefore, the expected Fd2 product should only have one observable intermolecular disulfide bond analogous to Fabs and as previously reported [14]. In Figure 2A, NR- and Red-cSDS profiles are shown for both HC- only Fd2-A and Fd2-B molecules that were C_H_1 affinity purified. For both molecules, Fd-only expression followed by C_H_1capture yields >98% purity by NR- and Red-cSDS. High SE-UPLC purities are also observed (>95%) that are easily polished by SEC purification to >99% SEC-UPLC purity like full-length HC2s (data not shown). Further, both molecules clearly form a covalent dimer by NR-cSDS, since a prominent peak at approximately 50 kDa is observed by NR-cSDS, and that this same molecule is reducible by DTT into monomeric Fd domains of approximately 25kDa for both Fd2-A and Fd2-B. The identity of these species was confirmed by intact mass spectrometry (data not shown). This demonstrated that the two different Fd2 species could be assembled, folded, secreted into the culture supernatant, and affinity captured followed by purification of the desired product without the presence of the Fc domain. This also revealed that the V_H_ and C_H_1 domains alone were sufficient to form a reducible covalent homodimer, without involvement by the Fc domain, where the two Fd domains are presumed to be linked by a covalent, intermolecular, and reducible disulfide bond. In Figure 2B, representations of the constructs evaluated thus far are shown and include a full-length mAb and HC2 as well as Fab and Fd2 domains. There, the Fd2 domains are shown to be associated biophysically and structurally as is later demonstrated.

### 3.7. Differential Scanning Calorimetry (DSC) Analysis of mAb, HC2, Fab, and Fd2 Forms

**Figure 3:**
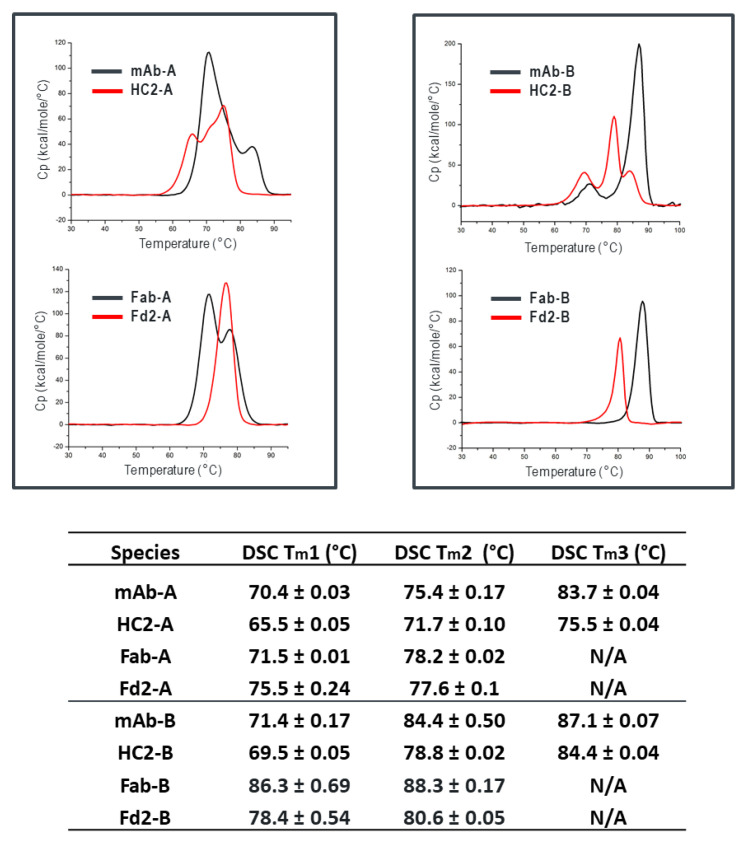
DSC profiles and tabulated T_m_ data for mAb, HC2, Fab, and Fd2 constructs for both A and B molecules. DSC profiles in the upper right box are for the A forms; upper left for the B forms. Below are the tabulated T_m_ values for each species.

Along with full-length mAb and HC2, corresponding Fab and Fd2 truncations were evaluated by Differential Scanning Calorimetry (DSC) to probe differences in thermal stability for both A and B molecular forms. In Figure 3, DSC profiles and tabulated T_m_ data are shown for mAb, HC2, Fab, and Fd2 forms. For both A and B, mAb and HC2 forms have similarly high, acceptable melting transitions and three in total for each molecule. For mAb-A and HC2-A, T_m_1 values of 70.4 °C and 65.5 °C were obtained, respectively. For mAb-B and HC2-B, T_m_1 values of 71.4 °C and 69.5 °C were obtained, respectively. For the Fab and Fd2 constructs, both A and B molecules also had similar and high melting transitions (two transitions for each molecule). Fab-A and Fd2-A T_m_1 values were 71.5 °C and 75.5 °C, respectively, with the Fd2 (HC-only domain) having higher thermal stability versus the Fab. For the B forms, the Fab and Fd2 T_m_1 values were 86.3 °C and 78.3 °C, respectively. In the latter case, while the Fd2 construct had lower thermal stability than the Fab counterpart, it still possessed high thermal stability by DSC with a T_m_1 nearly 80 °C. It is important to note that these Fd2 molecules can be expressed and purified to a fairly homogenous state (See Figure 2). Overall, DSC measurements clearly show that HC2/Fd2 forms for two different antibodies have high thermal stability on par with conventional mAbs/Fabs.

### 3.8. Formation and Analysis of (Non-Covalent) Fd2 Molecules without an Intermolecular Disulfide Bond

**Figure 4:**
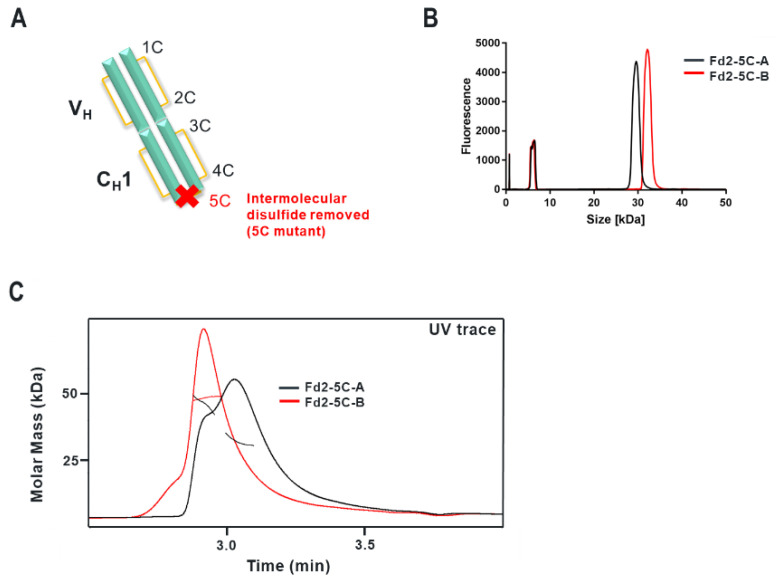
Representation of the Fd2-5C dimer variant and experimental data for both Fd2-5C-A and Fd2-5C--B molecules. (**A**) Representation of the dimeric Fd2 domain in cyan, with the V_H_ and C_H_1 regions labeled and segmented. Each disulfide bond is represented in yellow, and the location of each cysteine is labeled for one Fd domain (1C-5C counting from the N-terminus). The intermolecular disulfide bond connected by the 5C cysteines is removed by cysteine to serine mutagenesis and thus marked with a red “X”. (**B**) Overlaid NR-cSDS electropherograms for both Fd2-5C-A (black trace) and Fd2-5C-B (red trace) and (**C**) Overlaid SEC-MALS profiles for both Fd2-5C-A (black trace) and Fd2-5C- B (red trace). Results are plotted as molar mass (kDa) versus time with each chromatogram generated by UV absorption. Measured molar masses are overlaid on each chromatogram (Fd2-5C-A in black and Fd2-5C-B in red).

**Figure 5:**
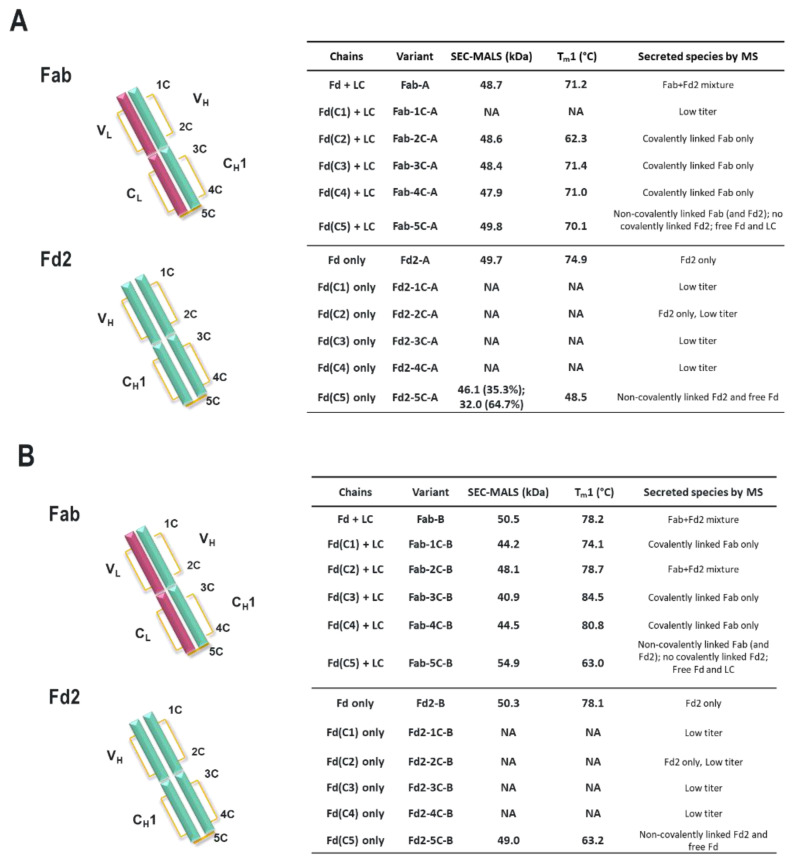
Diagrams of the Fab and Fd2 domains along with tabulation of transfected chains, variants, and SEC-MALS Mw and nano-DSF T_m_1 results for both molecules A (**A**) and B (**B**). The Fab domain is shown as the assembly of the LC (V_L_ + C_L_, red) and Fd (V_H_ + C_H_1, cyan). The Fd2 domain is also shown as a homodimer of Fd domains (cyan). Like in Figure 4A, disulfide bonds are depicted in yellow and the position of each cysteine is labeled 1C-5C counting from the N-terminus. Under the variant column is specified which cysteine is mutated to serine (e.g., Fab-1C-A has the first cysteine from N-terminus mutated to serine). Species identified by intact mass are listed. “NA” denotes no measurement since insufficient material was expressed and purified for characterization.

While the Fd domain forms a reducible, covalent dimer (Fd2) for two molecules with different HC framework germlines (HV1 and HV4 respectively) and CDR sequences that bind to two separate biological targets, we wanted to evaluate if these molecules could be expressed, secreted, and dimerized in solution without an intermolecular disulfide bond. Stoyle and coworkers reported that the 5th Cysteine from the N-terminus (Cys219 in Mab-A and designated herein as “5C”) is likely responsible for intermolecular disulfide formation between Fd domains [14]. This is the same HC cysteine residue responsible for forming the conserved Fab intermolecular disulfide between the HC and LC [27]. However, it is not known if this is conserved throughout observed HC dimers and further, if the Fd domains can self-dimerize non-covalently in solution. In other words, do Fd2 or HC2 dimers form a true dimerization interface in solution with substantial and relevant interface contacts that is apparent in mAb or Fab forms. When the corresponding 5C-Cys is mutated to serine for both molecules A and B (Cys219Ser in the case of mAb-A, diagrammed in Figure 4A), the Fd domain is still efficiently expressed and purified to high purity. Further, in Figure 4B, denaturing NR-cSDS analysis reveals an Fd monomer of approximately 25 kDa in size for both Fd2-5C-A and Fd2-5C-B, corresponding to the expected molecular weight. Therefore, removal of the disulfide originally responsible for HC-LC disulfide pairing in the Fab domain by “5C” cysteine to serine mutagenesis, also prevents the HC2 (Fd2) from forming a similar intermolecular disulfide bond. In Figure 4B, online SEC-MALS profiles are shown for both Fd2-A and Fd2-B with the 5C disulfide knock-out mutation (Fd2-5C-A and Fd2-5C-B), which elute and are detected from the sizing column at low μM concentration. In solution, a partially resolved mixture of monomeric Fd and non-covalent Fd2 is apparent for Fd2-5C-A. For Fd2-5C-B, non-covalent dimeric Fd2 species are predominant at an average Mw of 49.0 kDa, therefore indicating a more stable non-covalent dimer. We believe these dimeric species do not represent aggregated species, especially since previous accelerated stability work with HC2-A does not result in appreciable aggregation by SE- UPLC even after 1-month storage at 40 °C (See Table 4). Because the monomeric and dimeric species for Fd2-5C-A are not fully resolved, the measured Mw values will be an overestimation for the monomeric form and underestimation for the dimeric form and are tabulated in Figure 5.

### 3.9. Effect of Disulfide Mutagenesis on Fab and Fd2 Formation and Thermal Stability

Subsequently, we wanted to evaluate the effects of all disulfides in the V_H_ and C_H_1 regions on Fd2 expression and stability for both A and B molecules. Therefore, each V_H_ and C_H_1 cysteine was individually mutated to serine for both Fab and Fd2 constructs on both A and B molecules. Based on known IgG1 disulfide architecture [27], Cysteines 1-4 (for mAb-A, sequential numbering is Cys22 corresponds to “1C”, Cys96 is “2C”, Cys143 is “3C”, Cys199 is “4C”) form a pair of conserved intramolecular disulfides within the Fd portion of the Fab domain and pair as 1C-2C and 3C-4C respectively. The 5th cysteine from the HC N-terminus (Cys219 for mAb-A and Cys230 for mAb-B, or “5C”) forms the intermolecular disulfide between the Fd and LC of the Fab domains as aforementioned. These molecules will be designated as the construct format, followed by location of cysteine mutagenesis, followed by A or B molecule (e.g., “Fd2-2C-A”). In Figure 5A,B, SEC-MALS and nano-DSF results, along with species observed, are tabulated for each of the resulting variants. Nano-DSF was utilized here to evaluate T_m_ in a more high-throughput manner for many variants and generally yields results comparable to DSC. In WT Fab expression of both A and B molecules, a mixture of Fab and Fd2 species are observed. Since both Fab and Fd2 are ~50Kda in mass, SEC-MALS measured the Mw to be ~50KDa for this mixture for both A and B. For both Fab-A and Fab-B, Fab expression occurred for nearly all cysteine mutants with the exception of low Fab-2C-A expression. For Fab 1C-2C mutants, similar or lower T_m_1 values are observed by nano-DSF analysis, while similar or higher T_m_1 values are observed for 3C-4C mutants. SEC-MALS results for WT Fab and each of the 1C-4C Fab variants indicate that an intact Fab is expressed and formed as expected, with a measured Mw of ~50 KDa. When the intermolecular Fab disulfide is broken through 5C mutagenesis, T_m_1 is similar for Fab-5C-A but reduced ~15 °C for Fab-5C-B. These Fab-5C species are intact, non-covalent Fabs indicated by SEC-MALS and NR-cSDS results (intact Fab by native SEC-MALS and composed of monomeric Fd and LC components by denaturing NR-cSDS). When the same mutagenesis approach is applied to HC or Fd-only expressed molecules A and B, similar traits are observed with one notable exception: when any cysteine responsible for intramolecular disulfide formation is mutated in HC-only Fd2 expression, little or no protein expression is observed, and therefore no analysis was conducted. Only for the case of Fd2-2C-A expression was Fd2 even detectable by NR- cSDS. This explains why the same variants also resulted in pure Fab species for the Fab-A and Fab-B 1C-4C mutants, since 1C-4C mutated Fd2 was unable to be expressed to appreciable levels. Similar to the case of the Fabs, when the intermolecular disulfide bond is disrupted by 5C mutagenesis, intact, non-covalently dimerized Fd2 is expressed and purified as revealed by SEC- MALS and NR-cSDS (See also Figure 4). For both Fd2-5C-A and Fd2-5C-B, T_m_1 is significantly reduced compared to Fd2-A and Fd2-B, respectively. For Fd2-5C-A, T_m_1 is reduced 26 °C, and for Fd2-5C-B, T_m_1 is reduced 15 °C. This is similar to the case of the Fab-5C-B molecule, where T_m_1 is reduced 15 °C when the Fab domain is no longer held together non-covalently.

### 3.10. Crystallization and Structural Evaluation of Fd2-A

**Figure 6:**
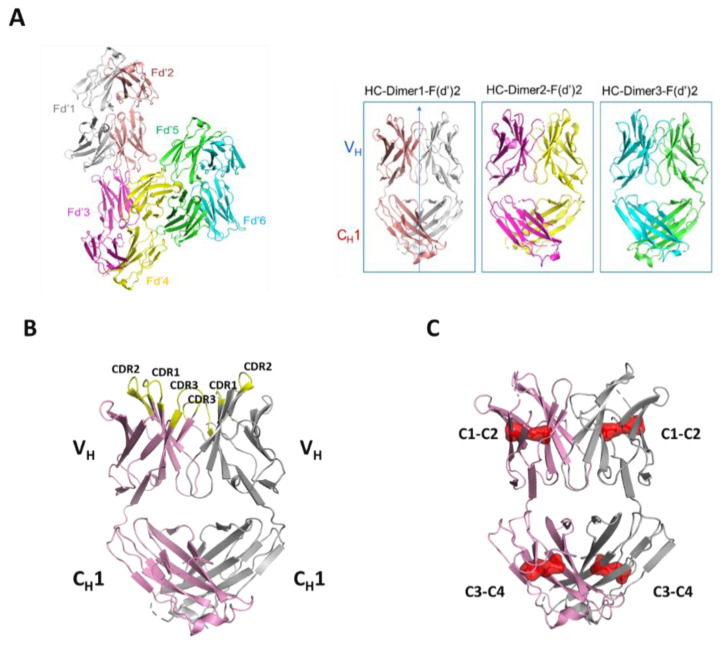

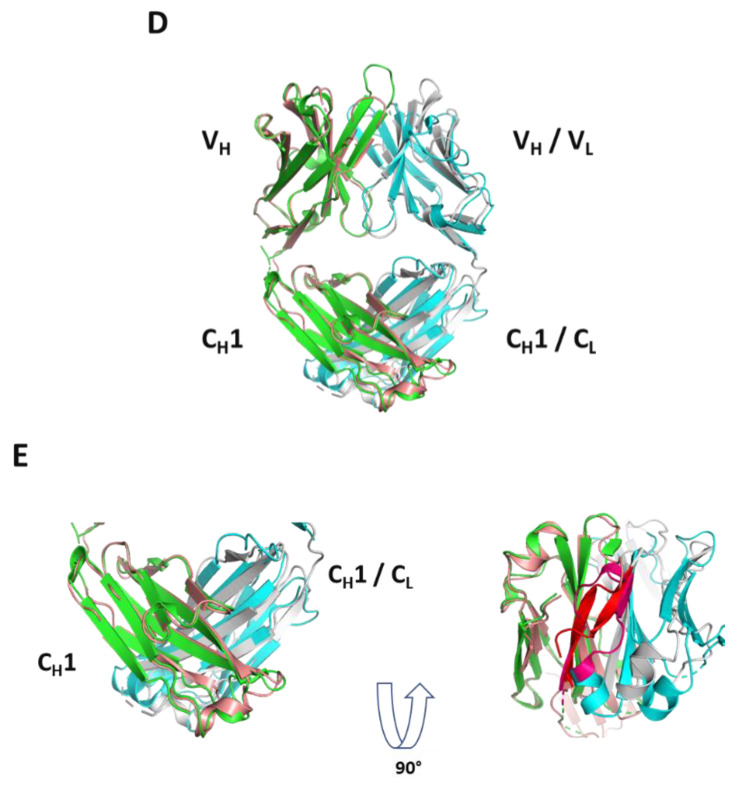
(**A**) Three HC or Fd dimers were found in asymmetric unit. All 3 dimers are similar and have the same topology except for minor differences in electron density in flexible regions. “HC- Dimer1-Fd2” (dimer of Fd’1 and Fd’2 domains) was chosen for evaluating structural features such as interfacial contacts, CDR topology, and comparisons to a representative Fab domain. (**B**) Ribbon diagram of the highly symmetrical HC dimer Fd2-A (PDB accession code 7KQY). One Fd chain is colored light purple and the opposing chain is gray. CDR loops are yellow and labeled. This representation is the same HC dimer as “HC-dimer1-Fd2” shown in part A. (**C**) Fd2-A with each intramolecular disulfide bond highlighted red in transparent surface and labeled with the same notation used in Figure 5 (C1, C2, etc., where C1 is disulfide bonded to C2 and C3 bonded to C4) (**D**) Superposition of Fd2-A dimer (shown as salmon for chain A and gray for chain B) and HC-LC of a Fab (PDB accession code 5vsi). The Fab HC (or Fd) chain is in green and the LC is in turquoise. (**E**) Superposition of the Fd2 C_H_1 domains with 5vsi Fab C_H_1 and C_L_ domains (left) and same domain rotated back 90° (right). In the HC2-A dimerization interface, residues from C_H_1 shown in red are residues HC-126 to HC-133 involved in a symmetrical interaction that is unique relative to the Fab interface. In magenta, this portion of the Fab HC-LC interface (residues HC-131 to HC-138 for 5vsi) are interactions not present in the HC2-A interface.

To understand the structural basis of HC dimer formation and the nature of HC dimerization, crystallization studies were conducted for the Fd2 domain of molecule A (Fd2-A). Crystals obtained from a sparse matrix screen diffracted to 2.9 Å and the structure was solved by Molecular Replacement (See Table 5 for crystallographic statistics). The resulting model was refined and built several times and led to final values of R_free_ and R_work_ of 26.8% and 19.8%, respectively. The final model consists of six monomers of 215 residues each that form three homodimers of HC:HC (Figure 6A) in the asymmetric unit. This is deposited in the PDB bank as PDB I.D. 7KQY. In the crystal structure, the three molecules within the asymmetric unit are structurally similar but have distinct and different domain orientations. The final electron-density map allowed the positioning of most residues with confidence. In Figure 6B, the Fd2-A structure is shown in ribbon diagram and reveals a highly symmetrical homodimer. The CDR loops are also highlighted and face outward, similar to how CDR loops are positioned in Fabs/mAbs. However, we found these HC2s for molecules A and B to not bind their original targets as expected using a BIAcore SPR affinity assay (data not shown). The electron density corresponding to residues HC-216 to 224 is very weak, indicating that the loop is disordered. This disordered loop also includes Cys219, which is engaged in an intermolecular disulfide bond to itself in the opposing monomer based on previous studies of HC dimers [14] and our disulfide mutagenesis experiments. While this loop is disordered and therefore not shown, it appears to be in close proximity to the same loop in the opposing Fd monomer, thus allowing for intermolecular disulfide formation. In Figure 6C, the Fd2-A homodimer is shown with all observed intramolecular disulfides shown in red surface. These pairs of intramolecular disulfides are conserved with respect to all IgG1 mAbs. In Figure 6D, the novel Fd2-A complex is superposed with a Fab whose crystal structure was solved and previously reported (PDB ID 5vsi). Specifically, the C_H_1 and V_H_ domains were superposed and overlay closely, with a low overall Cα RMSD of 1.10, highlighting how similar the HC2 (or Fd2) and Fab structures are. In Figure 6E, the C_H_1 domains of the Fd2-A and Fab (5vsi) are superposed. Interfacial Fd2-A dimer contacts that differ from 5vsi are shown in red, which includes symmetrical interactions involving the loop containing residues HC-126 through HC-133. Also in Figure 6E and overlaid with Fd2-A, the corresponding Fab loop (HC-131 to HC-138) is highlighted in magenta and is involved in separate interactions with the LC; it is in this region where a different set of unique interactions exist when comparing the Fd2-A and Fab structures (See Supplemental Figure S1 for sequence alignment).

**Figure 7:**
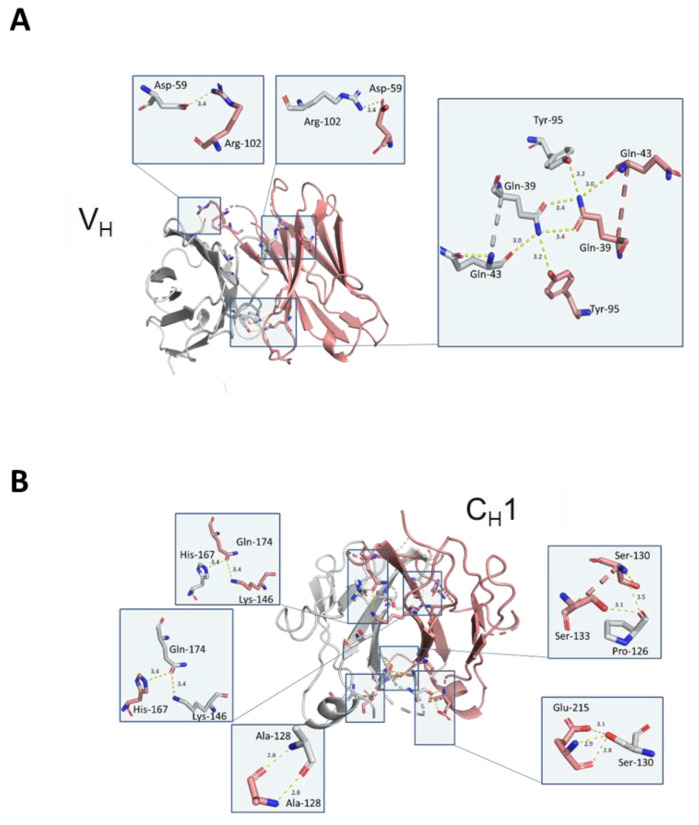
(**A**) Key interfacial contacts highlighted in the variable region (V_H_). (**B**) Key interfacial contacts highlighted in the constant region (C_H_1). Fd chains A and B are colored salmon and gray.

In the highly symmetrical Fd homodimerization interface, several key and conserved residues are apparent and hold the dimerization interface together (Figure 7A). Involved are residues spanning the V_H_ (Figure 7A) and C_H_1 (Figure 7B) domains and include both highly conserved FW and variable residues. In the FD2-A structure, the opposing C_H_1 domains are in direct contact with each other and the V_H_1 domains are in direct contact as well, analogous to the Fab structure. Between chains A and B (opposing monomer units within dimer) within the C_H_1 regions, key intermolecular contacts include side chain contacts between A-His-167 to B-Gln-174 (3.4 Å interatomic distance), side chain contacts between A-Gln-174 to B-His-167 (3.4 Å), backbone contacts between A-Ala-128 and B- Ala-128 (2.8 Å), side chain contacts between A-Pro-126 to both B-Ser-130 and B-Ser-133 (3.5 Å and 3.1 Å respectively), and side chain contacts between A-Ser130 to B-Gln-215 (2.9 Å). Additionally, a symmetrical network of interactions exists between conserved V_H_ FW residues Tyr-95 and Gln-39 of both chains A and B, where each side chain is contacted at an interatomic distance of 3.2 Å, with Gln- 43 flanking and stabilizing this network of interactions. Gln-39 in both chains also have side chain contacts with each other at a distance of 3.4 Å. Because these residues are conserved, the decision was not to mutate these residues and evaluate their effect on HC dimer formation as they are conserved across all mAbs. In the variable region of Fd2-A, key, highly symmetrical contacts exist mainly between the HC-CDR2 and HC-CDR3 regions (See Figure 7A). Hydrogens bonds are apparent between the side chains of residues HC-CDR2-A-Asp-59 and HC-CDR3-B-Arg-102 (3.4 Å distance), as well as HC-CDR3-A-Arg-102 and HC-CDR2-B-Asp-59 (3.4 Å distance).

Many of the apparently conserved interfacial homodimer contacts present in the Fd2-A structure have similar or analogous contacts present in the Fab (HC-LC) interface [28]. In 5vsi, intermolecular side chain contacts between the V_H_-V_L_ FWs are observed between HC-Gln-39 and LC-Gln-37, which appear to be substituted by a symmetrical HC-Gln-39 interaction in the Fd2-A structure. Additionally, the Fab HC-Tyr-95 to LC-Gln-37 interaction is replaced by duplicate, symmetrical HC- Tyr-95 to HC-Gln-39 interactions in the Fd2-A structure. In the Fab C_H_1-C_L_ interface, intermolecular side chain contacts are observed between HC-His-172 and LC-Asp-137. This appears to be replaced in the Fd2-A structure by a duplicate set of symmetrical HC-His-167 to HC-Gln-174 side chain interactions, where Fd2-A-His-167 aligns with His-172 in the 5vsi Fab. Towards the base of the Fd2-A complex, there is some divergence in contacts and structural alignment with 5vsi Fab, although there are additional contacts with residues in common to both Fd2-A and 5vsi Fab. For instance, in the Fab, an interfacial side chain contact exists between HC-Ser-138 (sequence aligns to Fd2-A-Ser-133) and LC-Phe-177.

### 3.11. Mutagenesis of Fd2-A V_H_ Interfacial Residues and Their Effect on HC2 Formation

**Figure 8:**
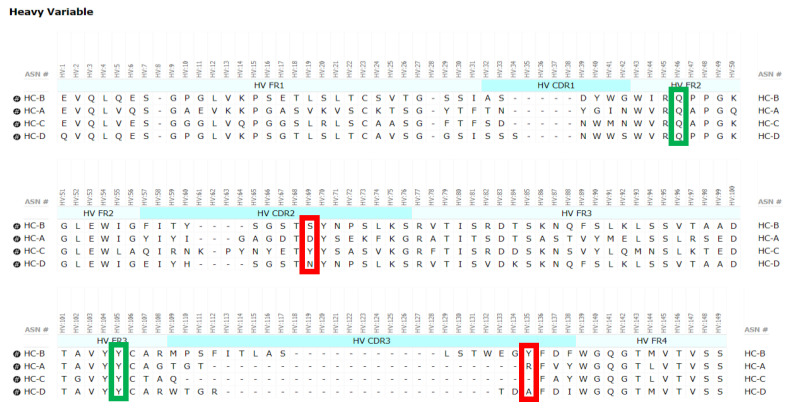
Sequence alignment of the V_H_ domain for the 4 molecules expressed at large scale, or HC- A, -B, -C and -D. Key conserved, interfacial HC2 V_H_ interactions are outlined in red/green. Variable residues where mutagenesis was attempted are indicated with a red box and involve aligned residues in the HC-CDR2 and HC-CDR3. Due to differences in HC-CDR3 lengths which are common, HC2-A R102 was aligned with HC2-C Q99, or the first HC-CDR3 residue. More conserved interfacial contacts where mutagenesis was not attempted are indicated in a green box.

Since the pair of aforementioned Fd2-A R102-D59 interactions are symmetrical between both chains in the homodimer and involve residues unique to that molecule, these residues were mutated to examine their effects on HC2 dimer formation. If these interfacial residues were mutated to different residues that do not favor HC dimer formation, the result should be a lower propensity to form HC dimer. These mutations either removed the side chain or were mutated to other residues present in other mAbs with little tendency to form HC dimer, specifically residues found in mAbs C and D (See Figure 8 for alignment). In Table 6, these results are summarized for % HC dimer formation for HC/LC cotransfection to produce mAbs in CHO transient cells. For mAb-A, HC-CDR2- D59N/A and HC-CDR3-R102A/Q mutations were engineered. For mAb-A, all of these mutations reduced the % HC dimer expressed and purified by several fold (in the range of 7 to 16.3 fold reduction). Further, using a sequence alignment of the V_H_ domains of both molecules A and B (Figure 8), the residues HC-S58 and HC-Y133 in mAb-B aligned with HC-D59 and HC-R102 in mAb- A, respectively. When similar mutations were made to these residues in mAb-B, namely HC- S58A/Y and HC-Y113A, the % HC dimer expressed and purified was reduced in the range of 6.9 to 27.5 fold. Remarkably, all these mAb variants maintained target binding in the sub-nM range similar to WT using a monovalent BIAcore SPR affinity assay against their respective target antigens (Table 6). Therefore, the Fd2-A crystal structure guided mutagenesis of both mAb-A and mAb-B, which in each case resulted in decreased HC dimer formation propensity. From a developability context, this indicates HC2 formation can be mitigated by site-directed mutagenesis of these sites while not impacting activity; from an engineering perspective to create HC2 molecules, similar reverse mutagenesis may be successful at similar or distinct sites.

## 4. Discussion

Herein, we structurally and biophysically characterized unexpectedly occurring human heavy- chain only antibodies arising directly from transient CHO expression. We found these non- artefactual HC-only antibodies to be homodimeric in nature, with an extended interface joining both C_H_1 and V_H_ domains, and to be linked by a conserved intermolecular disulfide bond similar to how Fabs are linked. Overall, these HC dimers are efficiently expressed with or without the LC and conserved Fc domain. Further, these HC-only molecules have similar or better accelerated storage stability characteristics and thermal stability compared to their mAb or Fab counterparts. Further, the HC dimer is structurally analogous to the Fab, and this is an interesting result that has implications concerning its mechanism of assembly and its potential application as a novel antibody format. The solved crystal structure and structure-guided, rational mutagenesis to the dimer interface reveals for the first time structural and sequence determinants to human heavy-chain only antibody formation.

For both mAbs A and B presented in this study, which are two humanized and functional recombinant antibodies with different germlines (HV1/KV2D and HV4/KV1 respectively) against different and unrelated biological targets, HC-only expression yields stable HC dimers (HC2s). These HC2s are reducible into monomeric components as demonstrated by denaturing R-cSDS of Fd2 forms and NR-cSDS of C219S-Fd2 forms (lacking the intermolecular disulfide, see Figure 2 and Figure 4). In Table 1, we see that expression variables and isotype do not eliminate or significantly impact HC dimer formation for molecule A (HC2-A), and HC dimer remains robust, although varied, across conditions. The only exception to this is with the addition of 5X transfected LC, which can effectively eliminate HC2-A formation but was not considered to be a viable downstream process solution.

Specifically, the HC cysteine involved in the intermolecular disulfide bond connecting each Fd domain in the Fd2 molecule is the same HC cysteine that disulfide links the Fd and LC together in Fabs. Interestingly, when the Fd-Fd (no Fc and hinge disulfides present) intermolecular disulfide bond is broken through C219S/C230S mutagenesis for Fd-A and FD-B respectively, these HC (Fd) dimers are still able to be expressed and yield molecules that are partially or predominantly non- covalently dimerized in solution at low μM concentrations. This was demonstrated by native SEC-MALS, suggesting there is a real dimerization interface with low μM affinity. This result also justified crystallographic studies in an attempt to structurally reveal the nature of this dimerization interface. This same experiment was conducted for Fab expression (HC or Fd +LC), however, since a mixture of non-covalent Fab and Fd2 was formed, it is difficult to parse out comparisons between non-covalent Fd2 and Fab properties.

Interestingly, while both IgG1 mAbs A and B efficiently yield HC dimer both in the full-length and Fd2 (without Fc) forms from HC-only CHO transient transfection, in HC/LC cotransfected CHO transient expression, HC2-B clearly is more efficiently expressed than HC2-A (46.2% vs. 4.9% in control tubespin cultures). While we deemed both of these mAb molecules to have huge developability risks and concerns due to robust HC dimer formation, mAb-B HC/LC transfection almost equally expressed HC2-B versus mAb-B. By SEC-MALS analysis, C230S-Fd2-B was also predominantly a non-covalently intact Fd2 dimer, whereas C219S-Fd2-A was predominately monomeric with some non-covalent dimer present. Considering the amount injected (20 μg) on the SE-UPLC, the measured UV signal, and the volume over which the C219/230S-Fd2-A/B analytes eluted (0.25–0.5 mL), these dimeric Fd2 species were detected by SEC-MALS in the 10–20 μM range. This same C230S-Fd2-B species had a higher T_m_1 (63 °C) than did C219S-Fd2-A (49 °C), although this difference may have been due to the instability of the predominant monomer form for C219S-Fd2-A at low μM concentrations. Empirically, it is evident that mAb-B had significantly higher levels of expressed HC2 in the presence of LC than did mAb-A and it also qualitatively has a stronger dimer interface in the solution state as suggested by SEC-MALS data. The stronger dimer interface present in Fd2-B versus Fd2-A may explain why Fd2-B forms more robustly in the presence of LC, where apparently HC2 or Fd2 formation is seemingly in direct competition with mAb and Fab formation and is more favored if the Fd2 interface is strengthened. Conversely, when mutations were made to the dimer interface elucidated by the novel Fd2 crystal structure that were predicted to weaken the dimer interface for both HC2-A and HC2-B, HC dimer levels decreased in the presence of LC expression. Therefore, these dimer interface mutants selectively decreased HC dimer formation versus competing HC-LC formation as originally hypothesized.

Conversely, if HC-LC pairing were strengthened or weakened, this should lower or increase HC dimer formation, respectively. When we express mAb-A in the presence of 5X LC, mAb expression dominates and we do not obtain any measurable amounts of HC2-A. This is a clear indication that mAb assembly outcompetes potential HC2 assembly when LC is expressed in vast excess. Moreover, when we evaluate HC/LC germline pairing frequencies, we see that uncommon germlines may increase the likelihood of HC2 formation (Table 2). In a publication by Jayaram and coworkers, 358 HC/kappa-LC human antibody pairings were evaluated, and specific germline frequencies were tabulated [25]. Their findings suggest that germline pairings are not random and that common germline pairings may be attributable to increased stability of the V_H_/V_L_ interface. In mAb-A, the HC and LC germlines are the result of a more uncommon germline pairing, HV1/KV2D, where only four instances out of 358 occurred. However, this uncommon pairing may not be statistically significant when considering that KV2D itself is infrequently encountered. Likewise, mAb-B also has an uncommon germline pairing, HV4/KV1 (nine out of 358 instances). When 11 additional human/humanized recombinant IgG1 and IgG4 mAbs against different targets were evaluated, nine had more common pairings (>20 instances each) and none formed HC dimers appreciably. Although our panel of molecules is limited and anecdotal, our findings may indicate germline pairing preferences play a role in enabling HC dimer formation and also explain their uncommon occurrence. However, clearly there are other intrinsic factors to HC dimer formation which are presumably sequence/structurally dependent. The heavy-chain domain still needs to be formed, folded, and result in a reasonably stable product independent of the LC, a characteristic unique to a certain subset of human HC sequences.

Another example or factor in HC2 formation may include the length of the HC-CDR3, where HC2-B has an unusually long HC-CDR3 of 19 residues (See Figure 8). HC-CDR3 length and composition can have a huge impact on the developability properties of a mAb [29]. Previously, HC-CDR3 length has correlated with negative developability attributes such as aggregation and high viscosity, where 137 clinical-stage antibody therapeutics had a median HC-CDR3 length of 12 residues [30]. For HC/LC transfection of mAb-B, HC2 formation (46.2%) was nearly equal to normal mAb assembly (See Table 2). Stoyle and coworkers reported the number of basic residues to be a determinant of HC2 expression, where an increase in basicity of the HC-CDR3 increased HC2 propensity [14]. However, no basic residues are observed in the HC-CDR3 for HC2-B. In the case of HC2-A, a relatively short HC-CDR3 of seven residues exists, with only one basic residue present. Clearly, HC2 formation may depend on other complex and unknown antibody sequence and structural factors.

It is well known that the molecular chaperone protein, BiP, binds non-covalently to the HC, but not to the HC associated with LC [31]. During mAb assembly, HC-LC pairing and folding in the Fab arm occurs following BiP release from unfolded HC (C_H_1 + V_H_ or Fd) as a result of LC assembly [32]. Here in the case of molecules A and B, HC dimer formation occurs with or without the presence of LC, implying that perhaps one Fd domain mimics the LC, and can promote folding of another Fd domain presumably associated to BiP. Remarkably, as depicted in Figure 6D, the novel HC2 or Fd2 complex reported herein overlays nicely with a representative Fab structure with a low overall Cα RMSD of 1.10 Å, revealing that the opposing HC, or Fd, likely mimics the LC structurally during assembly and promotes complete folding and assembly of HC2s/Fd2s. While it is known that HC dimers are common degradant products of formulated mAb (HC2-LC2) drug products [33], resulting from the loss of two LC domains, these HC2s described herein form during expression without LC, and are secreted and captured as fully folded and stable products. Indeed, we show that these novel HC-only antibodies are highly stable with T_m_s similar or better than their mAb or Fab versions (see Figure 3), where specifically Fd2-A had a measured T_m_1 of 75.5 °C compared to 71.5 °C for Fab-A. Additionally, we found that full-length HC2-A had excellent accelerated storage stability attributes that were superior to mAb-A, where after 1-month storage at 40 °C, purity attributes by SE-UPLC and NR-cSDS were both >98% (See Table 4).

It is also noteworthy that intramolecular disulfide formation is necessary for HC2 or Fd2 expression. When one intramolecular disulfide bond is removed in the Fab-A and Fab-B forms, LC assembly followed by BiP removal is still able to occur, and Fab folding and secretion is achieved. The result for Fab-A and Fab-B are expressed Fab molecules with suitably high T_m_ values. This is not unexpected since reduced disulfides can occur naturally in antibodies [34], although incomplete disulfide formation may result in increased aggregation propensity or decreased bioactivity [35,36,37]. In HC2/Fd2 formation, one Fd domain appears to mimic or replace the role of the LC, however, when one intramolecular disulfide is removed on both Fd chains via mutagenesis, Fd2 assembly is not efficient and little to no product is yielded. This demonstrates that complete disulfide formation is necessary in one or both Fd domains to assemble to the Fd-BiP intermediate(s) and complete Fd2 folding and BiP release. While mutagenesis studies of C_H_1 cysteines involved in both inter- and intramolecular disulfide formation for a mouse IgG2 enabled expression and secretion of HC-only transcripts [38], we find complete intramolecular disulfide formation a requirement for the expression of two different humanized HC-Abs evaluated in this study.

Structurally, the solved crystal structure of Fd2-A is remarkably symmetrical and involves many conserved residues in the homodimer interface that have similar or analogous interactions in the Fab HC-LC interface. Like Fabs, the CDR loops are facing outward and primed for and amenable to antigen binding. As aforementioned, our novel HC-only Fd2 complex superposes remarkably with a representative Fab structure (5vsi, See Figure 6D), demonstrating that these molecules are quite similar to Fabs, and therefore have potential therapeutic utility. Although HC dimers A and B characterized in this study are not active against the same target antigens that the mAb/Fabs are (both in the sub-nM affinity range), this is fully expected since the entire LC is missing and further, the arrangement and conformation of the HC-CDR loops are very likely to differ between the HC2 and mAb forms. In the typical Fab structure, principal contacts exist between the HC-CDR3 and LC- CDR1. In this HC2 structure for molecule A, the HC-CDR3 is also primarily involved in interfacial contacts, with key contacts existing between the opposing HC-CDR2 chain, which apparently substitutes for the LC-CDR1 in mAbs. The HC2 homodimer structure is also highly symmetrical in nature, which may offer interesting binding and therapeutic properties, and may perhaps be amenable to engineering hetero-HC dimers (composed from two different HCs) that have functional utility. To accomplish this, HC-only domain libraries could be selected against a given target by yeast surface display, as many non-conventional antibodies and scaffold proteins have been engineered using this platform technology [39]. Further, hetero-human-HC dimers may be engineered to associate orthogonally by utilizing interfacial knob-hole interactions, for instance. These approaches may facilitate multi-specific formats in the presence of conventional Fab domains.

Mutations designed to disrupt the Fd2 dimer interface were based on direct V_H_ contacts revealed by the crystal structure of Fd2-A. These mutations were made for mAb-A and to aligned residues in mAb-B. When these key, interfacial CDR residues present in Fd2-A are mutated to smaller residues or residues present in other non-HC dimer forming mAbs (C and D), HC dimer expression is significantly reduced for both A and B (See Table 6). Likewise, the HC2-A and HC2-B molecules have many other similarities, such as both being linked by an intermolecular disulfide bond and having high thermal stability, indicating that the structures of HC2-A and HC2-B are likely similar and conserved. Additionally, as demonstrated by mutagenesis of interfacial dimer contacts, further engineering is achievable to modulate HC dimer formation through engineering or display technologies. Surely, other sequence or specificity determinants of HC2 formation likely exist and understanding these will further advance potential engineering. The use of engineered and/or antibody generated human heavy-chain dimers can have advantages such as being smaller in nature versus full-length antibodies as well as avoiding LC mispairing in multi-specific formats [40]. We show herein these human HC dimers are highly stable, structurally well-ordered, crystallizable, and their likely conserved and modular nature presents a path forward towards potential engineering and development of these molecules into therapeutic modalities.

## 5. Conclusions

The novel characterization and structural elucidation of dimeric and highly symmetric human heavy chain-only dimers (HC2s) were revealed for the first time and have significant implications to both the mechanism of antibody assembly and their potential application as a novel and functional antibody format. Unlike mAbs or Fabs, these HC2 species are expressed efficiently, independent of the LC, where one HC domain seemingly mimics the LC during assembly. These novel HC2s structurally overlay closely with Fabs, while sharing the same Fab disulfide bond network, secondary folds, and residues involved in their respective HC2 dimer or Fab interfaces. These HC2 species can also be purified to high homogeneity and are highly stable with thermal and acceleration storage stability similar or greater than their mAb or Fab counterparts. We verified residues in both the HC- CDR2 and HC-CDR3 in two different monoclonal antibodies against different targets to dramatically affect HC dimer propensity, highlighting for the first-time distinct sequence specificity determinants for this novel modality. We also elucidated important developability considerations and how to avoid HC2 formation during mAb expression, both from a sequence evaluation and process standpoint. Like Fabs, these molecules also present the CDRs outward for potential antigen binding, which can be exploited to find binding partners and potentially have utility as a novel antibody format.

## Figures and Tables

**Table 1:** Expression variables, codon usage, and isotype evaluated for their effects on mAb-A HC2 formation for 10 mL small-scale transient cultured CHO cells. Yield in milligrams refers to the amount of mAb expressed and affinity captured by ProA except for the HC-only transfection, where yield is the amount of HC2 expressed and purified. Control IgG1 mAb did not yield measurable amounts of HC2 with both HC-only transfection and HC/LC cotransfection.

Culture Conditions	Yield (mg)	%HC2 by NR-cSDS	Yield (mg)	%HC2 by NR-cSDS	Yield (mg)	%HC2 by NR-cSDS
	IgG4 mAb-A		IgG1 mAb-A		Control IgG1 mAb	
**HC only Transfection**	1.0	99.7	1.3	98.0	0.0	0.0
**Co-transfection with LC**						
Tube spin control	2.5	7.4	2.8	4.9	1.9	0.0
5x LC chain	2.1	0	1.8	0	N/A	
20% coding DNA	1.9	2.8	1.6	15.7	N/A	
Day 4 harvest	0.5	3.5	1.3	2.0	N/A	
No enhancer	2.8	2.6	2.2	2.6	0.3	0.0
No Temperature shift	0.9	2.3	1.0	2.7	0.5	0.0
Shake flask	2.3	5.0	2.4	2.4	2.1	0.0
Codon optimization 1	3.0	3.5	N/A		N/A	
Codon optimization 2	0.1	1.6	N/A		N/A	

**Table 2:** For a panel of 13 recombinant antibodies, tabulated are the HC/LC germlines. These are highlighted red if considered uncommon (<10 occurrences) and unhighlighted if common (>20 occurrences out of 358 kappa-LC containing sequences) [25]. Also tabulated are the % HC dimer (HC2) quantified by NR-cSDS from HC/LC cotransfection (mAb production) and the amount in mg of HC2 species obtained from HC-only small-scale transfection (10 mL total volume).

mAb	HC/LC Germlines	% HC2	HC-Only (mg)
**A**	HV1/KV2D	4.9	1.3
**B**	HV4/KV1	46.2	1.5
**C**	HV3/KV3	<0.1	<0.1
**D**	HV1/KV2D	<0.1	<0.1
**E**	HV1/KV1	<0.1	<0.1
**F**	HV3/KV1	<0.1	<0.1
**G**	HV1/KV1	<0.1	<0.1
**H**	HV1/KV1	<0.1	<0.1
**I**	HV3/KV1	<0.1	<0.1
**J**	HV4/KV2	<0.1	<0.1
**K**	HV1/KV1	<0.1	<0.1
**L**	HV3/KV1	<0.1	<0.1
**M**	HV3/LV3	<0.1	<0.1

Highlights denote infrequent occurance.

**Table 3:** Yields and SE-UPLC, NR-cSDS, and Red-cSDS purities tabulated for four HC2s expressed as HC-only transient transfections at 3 L scale followed by ProA affinity capture.

Molecule	Yield (mg/L)	% SE-UPLC Purity	% NR-cSDS Purity	% R-cSDS Purity
**HC2-A**	61	88.2	93.8	97.4
**HC2-B**	103	84.3	91.2	96.5
**HC2-C**	5	80.6	92.6	92.2
**HC2-D**	0.7	72.8	67.8	66.5

**Table 4:** Formulation conditions were 5 mg/mL for both mAb-A and HC2-A in 20 mM sodium acetate pH 5.5, a representative formulation condition. Both samples were stored up to 4 weeks at 40 °C. Column representations are % purity by NR-cSDS, % purity by SE-UPLC, and % High Molecular Weight species (HMW) quantified by SE-UPLC. Molecules were purified by ProA affinity chromatography followed by SEC polishing.

Molecule	Timepoint	NR-cSDS	SE-UPLC	% HMW
**mAb-A**	Initial	99.1	99.6	0.4
	2 wk 40 °C	97.8	99.2	0.4
	4 wk 40 °C	96.1	98.7	0.5
**HC2-A**	Initial	100	99.7	0.3
	2 wk 40 °C	99.5	99.5	0.5
	4 wk 40 °C	98.1	99.2	0.8

**Table 5:** Crystal data collection and refinement statistics. Values in parenthesis describe the highest resolution shell. Ramachandran refers to backbone dihedral angles.

*Data Collection*	HC2-A (7KQY)
Space group	C121
*Unit cell dimensions*	
*a*, *b*, *c* (Å)	169.66, 73.42, 140.41
α, β, γ (˚)	α = 90, β = 125.71, γ = 90
Resolution (Å)^a^	114–2.91 (3.02–2.91)
Unique reflections	22,757 (131)
Completeness (%)	73.43 (4.28)
Multiplicity	3.4 (1.7)
Wilson B-factors	54.5
*I*/σ*I*	4.85 (1.18)
*R*merge	0.103 (0.471)
*R*meas	0.146 (0.665)
*R-pim*	0.103 (0.471)
CC1/2	0.979 (0.619)
CC *	0.995 (0.874)
***Refinement***	
Resolution (Å)	114–2.91 (3.02–2.91)
Unique reflections	22,743 (131)
*R*work/*R*free (%)	19.8/26.8
CC work/CC free	0.933/0.905
Atoms	9247
Protein	9247
Protein residues	1243
RMSD Bond lengths (Å)	0.01
RMSD Bond angles (˚)	1.28
Ramachandran preferred/allowed (%)	88.2/8.7
Clashscore	10.84
Average B-factor	41.4

**Table 6:** For mAb HC/LC cotransfection, effect of HC2 formation for both mAb-A and mAb-B is evaluated for each mutant predicted to reduce HC dimer propensity by the Fd2-A crystal structure. % mAb form and % HC2 quantified by NR-cSDS are tabulated along with fold reduction compared to the wild-type version. Lastly, BIAcore affinities of the mAb (KD) towards recombinant target antigen as the analyte are tabulated.

Reagent Name	% HC2	HC2 Fold Reduction	% mAb	KD (nM)
**mAb-A WT**	**4.9**	-	**92.5**	**0.29**
mAb-A R102Q	0.5	9.8	**97.7**	0.3
mAb-A R102A	0.5	9.8	**98.0**	0.46
mAb-A D59N	0.3	16.3	**98.1**	0.67
mAb-A D59A	0.7	7	**97.7**	0.56
**mAb-B WT**	**46.2**	-	**47.4**	**0.11**
mAb-B Y113A	1.7	27.5	**90.7**	0.41
mAb-B S58Y	6.7	6.9	**87.9**	0.27
mAb-B S58N	6.6	7	**88.0**	0.23

## Supplementary Materials

The following are available online at https://www.mdpi.com/2073-4468/9/4/66/s1, Figure S1: HC2-A and HC-Fab Sequence alignment.
