# Supplementary material for: Crystal Structure and Characterization of Human Heavy-Chain Only Antibodies Reveals a Novel, Stable Dimeric Structure Similar to Monoclonal Antibodies
Source: Antibodies (Basel). 2020 Nov 22;9(4):66. doi: 10.3390/antib9040066 (PMC7709113; doi:10.3390/antib9040066)
Supplement: Supplementary file 1 [file antibodies-09-00066-s001.pdf]

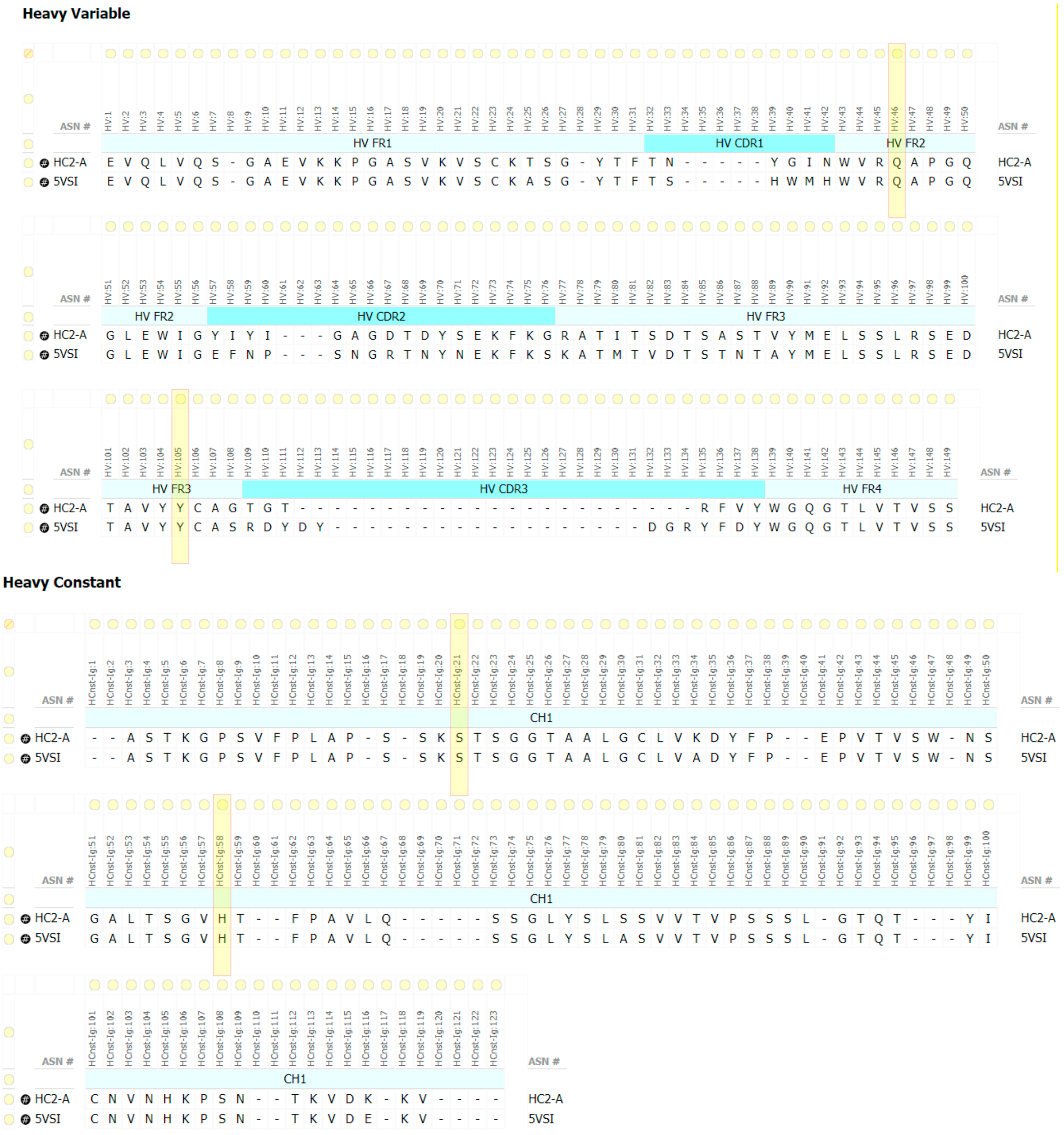

Figure S1. Sequence alignment for the heavy chain constant (CH1) and variable (VH) regions for HC2- A and 5vsi. Residues that engage in interfacial domain (CH1-CH1, VH-VH) contacts in common to both HC2-A and 5vsi are highlighted in yellow.
